# Sex-specific cardiac magnetic resonance pulmonary capillary wedge pressure

**DOI:** 10.1093/ehjopen/oeae038

**Published:** 2024-05-15

**Authors:** Pankaj Garg, Ciaran Grafton-Clarke, Gareth Matthews, Peter Swoboda, Liang Zhong, Nay Aung, Ross Thomson, Samer Alabed, Ahmet Demirkiran, Vassilios S Vassiliou, Andrew J Swift

**Affiliations:** Norwich Medical School, University of East Anglia, Norwich Research Park, Rosalind Franklin Road, Norwich NR4 7UQ, UK; Department of Cardiology, Norfolk and Norwich University NHS Foundation Trust, Colney Lane, Norwich NR4 7UY, UK; Norwich Medical School, University of East Anglia, Norwich Research Park, Rosalind Franklin Road, Norwich NR4 7UQ, UK; Department of Cardiology, Norfolk and Norwich University NHS Foundation Trust, Colney Lane, Norwich NR4 7UY, UK; Norwich Medical School, University of East Anglia, Norwich Research Park, Rosalind Franklin Road, Norwich NR4 7UQ, UK; Department of Cardiology, Norfolk and Norwich University NHS Foundation Trust, Colney Lane, Norwich NR4 7UY, UK; Leeds Institute of Cardiovascular and Metabolic Medicine, University of Leeds, Leeds, UK; National Heart Research Institute Singapore, National Heart Centre Singapore, 5 Hospital Drive, Singapore; Signature Programme of Cardiovascular Metabolic and Disorders, Duke-NUS Medical School, 8 College Road, Singapore; William Harvey Research Institute, NIHR Barts Biomedical Research Centre, Queen Mary University of London, London, UK; William Harvey Research Institute, NIHR Barts Biomedical Research Centre, Queen Mary University of London, London, UK; National Institute for Health and Care Research, Sheffield Biomedical Research Centre, Sheffield, UK; Department of Infection, Immunity & Cardiovascular Disease, University of Sheffield, Sheffield, UK; Department of Cardiology, Amsterdam Cardiovascular Sciences, Amsterdam UMC, Vrije Universiteit Amsterdam, Amsterdam, Netherlands; Department of Cardiology, Kocaeli City Hospital, Kocaeli, Turkey; Norwich Medical School, University of East Anglia, Norwich Research Park, Rosalind Franklin Road, Norwich NR4 7UQ, UK; Department of Cardiology, Norfolk and Norwich University NHS Foundation Trust, Colney Lane, Norwich NR4 7UY, UK; National Institute for Health and Care Research, Sheffield Biomedical Research Centre, Sheffield, UK; Department of Infection, Immunity & Cardiovascular Disease, University of Sheffield, Sheffield, UK; INSIGNEO, Institute for in silico Medicine, University of Sheffield, Sheffield, UK

**Keywords:** Sex, Heart failure, CMR

## Abstract

**Aims:**

Heart failure (HF) with preserved ejection fraction disproportionately affects women. There are no validated sex-specific tools for HF diagnosis despite widely reported differences in cardiac structure. This study investigates whether sex, as assigned at birth, influences cardiac magnetic resonance (CMR) assessment of left ventricular filling pressure (LVFP), a hallmark of HF agnostic to ejection fraction.

**Methods and results:**

A derivation cohort of patients with suspected pulmonary hypertension and HF from the Sheffield centre underwent invasive right heart catheterization and CMR within 24 h of each other. A sex-specific CMR model to estimate LVFP, measured as pulmonary capillary wedge pressure (PCWP), was developed using multivariable regression. A validation cohort of patients with confirmed HF from the Leeds centre was used to evaluate for the primary endpoints of HF hospitalization and major adverse cardiovascular events (MACEs). Comparison between generic and sex-specific CMR-derived PCWP was undertaken. A total of 835 (60% female) and 454 (36% female) patients were recruited into the derivation and validation cohorts respectively. A sex-specific model incorporating left atrial volume and left ventricular mass was created. The generic CMR PCWP showed significant differences between males and females (14.7 ± 4 vs. 13 ± 3.0 mmHg, *P* > 0.001), not present with the sex-specific CMR PCWP (14.1 ± 3 vs. 13.8 mmHg, *P* = 0.3). The sex-specific, but not the generic, CMR PCWP was associated with HF hospitalization (hazard ratio 3.9, *P* = 0.0002) and MACE (hazard ratio 2.5, *P* = 0.001) over a mean follow-up period of 2.4 ± 1.2 years.

**Conclusion:**

Accounting for sex improves precision and prognostic performance of CMR biomarkers for HF.

## Graphical Abstract

**Graphical Abstract:**
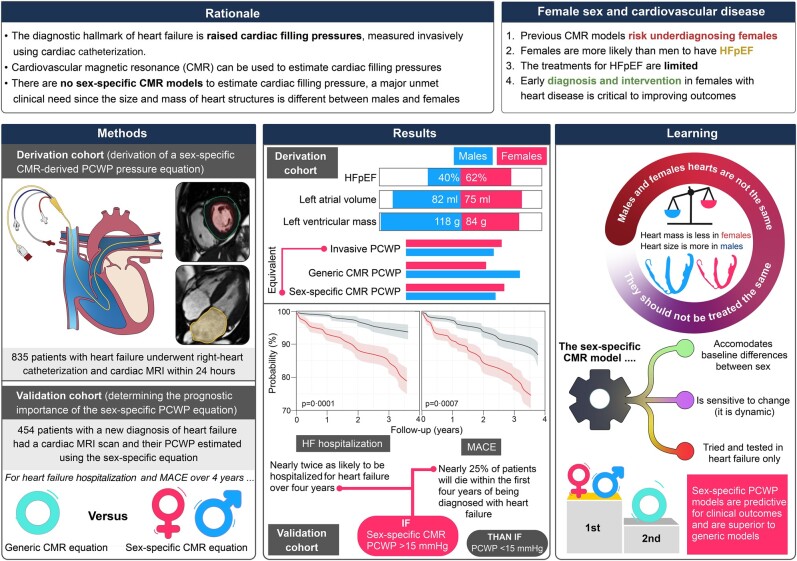

## Introduction

There is an urgent need for the development of distinct strategies to improve the diagnosis and treatment of heart disease in women.^1^ Heart failure (HF) is a growing global health concern with an estimated prevalence of over 64 million individuals worldwide.^2^ The exact number of these who are women is understudied and therefore uncertain. However, women suffer disproportionately from HF with preserved ejection fraction (HFpEF) due to risk factors such as aging and hypertension.^3^ The use of ejection fraction (EF) to classify HF sub-phenotypes is extensively adopted clinically but increasingly recognized to have significant drawbacks.^4^ The therapeutic options for HFpEF are therefore more limited than for HF with reduced ejection fraction (HFrEF),^5^ thus driving healthcare inequality. Women are less likely to be referred for specialist HF care^6^ or to receive optimized guideline therapy^7,8^ and, as a result, report lower quality of life independent of EF or natriuretic peptide level.^8,9^ Sex-specific differences in cardiac imaging^10^ and circulating biomarkers have previously been described.^11^

Heart failure is a clinical diagnosis made from clinical signs and symptoms, in the context of structural or functional cardiac impairment.^12^ Left ventricular (LV) filling pressure (LVFP) is a fundamental, physiological measure of HF, which directly drives congestive symptoms. The gold standard measurement of LVFP is invasively conducted during cardiac catheterization either directly as the LV end-diastolic pressure or indirectly during right heart catheterization (RHC) as the pulmonary capillary wedge pressure (PCWP). These methods are laborious and carry procedural risk to the patient, limiting their widespread adoption. Non-invasive estimation of LVFP is therefore crucial in the diagnosis and management of HF.^12^ While echocardiography remains the mainstay for non-invasive estimation of LVFP,^13^ cardiovascular magnetic resonance (CMR) has also recently been used.^14^ Cardiovascular magnetic resonance already plays an important role in sub-phenotyping HF,^15–17^ making it a useful non-invasive tool in the diagnostic workflow of all patients with suspected HF.

Cardiovascular magnetic resonance–derived PCWP has been shown to have prognostic utility.^14,18^ It is superior to transthoracic echocardiography in correctly classifying patients as having ‘normal’ or ‘raised’ filling pressures and can better determine the risk of cardiovascular death. Other large HF cohort studies have demonstrated the clinical significance of raised CMR-derived PCWP,^19^ showing that elevated CMR-derived PCWP is strongly associated with symptoms (orthopnoea, breathlessness) and signs of HF (pleural effusions, lower limb oedema). Furthermore, raised CMR-derived PCWP is independently associated with subsequent HF hospitalization and major adverse cardiovascular events (MACEs).^19^ In addition to its role in HF, CMR-derived PCWP can also be used to measure acute and dynamic changes in preloading conditions on the LV during adenosine-administered first-pass perfusion CMR, where it has been observed to rise significantly.^20^

The CMR-derived PCWP estimation has been independently shown to correlate well with invasively measured pressures, with good accuracy for HFpEF diagnosis; however, it may overestimate PCWP at rest and underestimate it during exercise.^21^ This highlights the need for refinement of the equation, and concerns have been raised regarding the use of LV mass (LVM) and left atrial volume (LAV) to estimate PCWP. Both these indices are sex dependent; hence, CMR-derived PCWP may overestimate PCWP in males and underestimate PCWP in females. This may affect the diagnostic precision of CMR-derived PCWP resulting in overdiagnosis of HF in men and under-diagnosis in women.

The aim of this study was to investigate whether CMR-derived PCWP is sex dependent, develop sex-specific CMR models to estimate PCWP, and to investigate whether a novel sex-specific CMR-modelled PCWP is prognostically significant.

## Methods

### Study population

In all participants, HF was diagnosed according to the European Society of Cardiology guidelines for HF^12^ and pulmonary hypertension.^22^ Specifically, this requires the presence of at least one symptom (e.g. breathlessness) and one clinical sign (e.g. peripheral oedema) of HF, as well as objective evidence of cardiac dysfunction (i.e. reduced EF or diastolic dysfunction on echocardiography). Two cohorts of patients were included: a derivation cohort (ASPIRE registry, Sheffield, UK) and a validation cohort (Leeds, UK). The ASPIRE registry is composed of patients with suspected pulmonary hypertension referred to Sheffield Teaching Hospitals NHS Foundation Trust, which includes all World Health Organization (WHO) groups, referred over 8 years (2012–20). Patient phenotype is extensively investigated using imaging and RHC measurements, with subsequent biomarker and pulmonary function investigations performed where appropriate. Right heart catheterization data allowed differentiation between WHO Group 2 pulmonary hypertension secondary to left heart disease and pre-capillary pulmonary hypertension, with CMR allowing investigation of cardiac dysfunction. The RHC and CMR were performed within 24 h, limiting haemodynamic changes. For the validation cohort, cardiology clinic patients with a new diagnosis of HF^12^ in the preceding 12 months were prospectively recruited (2018–20) and underwent CMR evaluation. Diastolic dysfunction was diagnosed as per the British Society of Echocardiography guidelines. Exclusion criteria were primary pulmonary arterial hypertension, significant valvular heart disease, and contraindications to CMR (e.g. inability to lie flat, claustrophobia, and end-stage HF). Sex was physician reported as a binary male or female category, referring to the sex assigned at birth rather than the gender identity or karyotype of the patient. Reporting adhered to the SAGER guidelines.^23^ The study was approved by Sheffield Teaching Hospitals and the UK National Research Ethics Service (16/YH/0352 and 17/YH/0300) and complied with the Declaration of Helsinki.^24^

### Invasive study

A balloon-tipped 7.5 French thermodilution catheter (Becton-Dickinson, Franklin Lakes, NJ) was used to perform the RHC. The PCWP was recorded using standard techniques and averaged over multiple cardiac cycles to avoid overestimating it.^25^ The recording was made when the patients were relaxed and had minimal beat-to-beat variation.

### Cardiovascular magnetic resonance study

**Figure 1:**
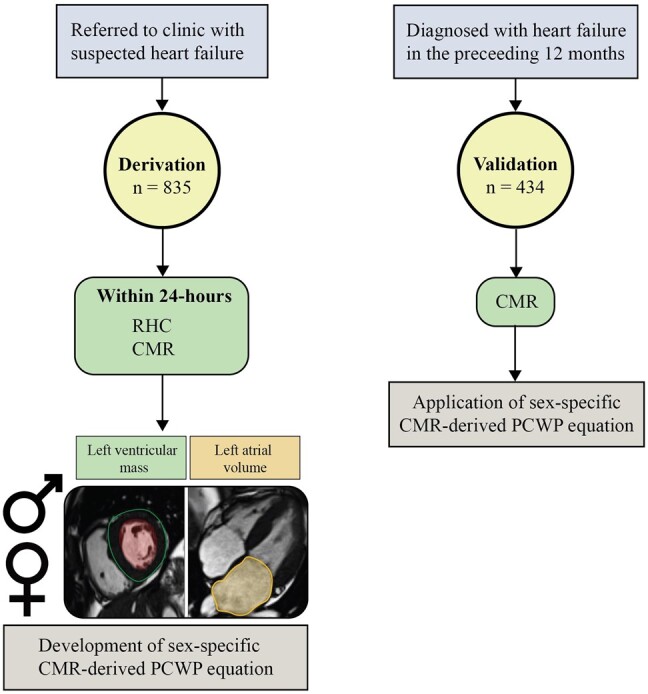
Study protocol. Within the derivation cohort, 835 subjects (Sheffield centre) were referred over an 8-year period (2012–20) for further assessment of breathlessness. Cardiovascular magnetic resonance and right heart catheterization were performed within 24 h of each other. A sex-specific cardiovascular magnetic resonance–derived pulmonary capillary wedge pressure equation was derived, which included left ventricular mass and left atrial volume as cardiovascular magnetic resonance metrics. Within the validation cohort, 434 subjects (Leeds centre) with a diagnosis of heart failure in the preceding 12 months underwent cardiovascular magnetic resonance. The sex-specific cardiovascular magnetic resonance–derived pulmonary capillary wedge pressure equations were applied. CMR, cardiac magnetic resonance; PCWP, pulmonary capillary wedge pressure; RHC, right heart catheterization.

For the derivation group, imaging procedures involved the utilization of a 1.5T whole-body GE HDx scanner (GE Healthcare, Milwaukee, USA), while the validation cohort utilized a 3.0T Siemens Magnetom Prisma scanner (Siemens, Erlangen, DE). Cine images were captured in four-chamber (4Ch), two-chamber (2Ch), three-chamber (3Ch), and short-axis (SA) orientations. This was achieved using a retrospective electrocardiogram-gated multi-slice steady-state free precession sequence, adhering to standard protocols (*Figure 1*).^26^ Blinded offline image analysis was conducted using either the GE Advantage Workstation 4.1 or Circle cvi42 (Circle Cardiovascular Imaging, Calgary, CA).

Manual delineation of endocardial and epicardial surfaces, excluding papillary muscles, was carried out on the stack of SA cine images. This facilitated the computation of various cardiac volumes, including LV end-diastolic volume (LVEDV), LV end-systolic volume (LVESV), right ventricular (RV) end-diastolic volume (RVEDV), and RV end-systolic volume (RVESV). Left ventricular stroke volume (LVSV), LVEF, RV stroke volume (RVSV), and RVEF were derived from these volumes using standard formulae. Left ventricular mass was derived using the SA cine stack at end-diastole. For left atrial assessment, the endocardium was contoured in both 4Ch and 2Ch views, enabling the determination of maximum LAV just before mitral valve opening (LV end-systolic phase) using the biplane area-length method.

### Outcome measures

For the validation cohort, patient outcomes were evaluated by reviewing electronic hospital records for MACE and hospitalization due to HF. Major adverse cardiovascular event was defined as the composite of cardiovascular death, HF hospitalization, non-fatal stroke, and non-fatal myocardial infarction.

### Statistical analysis

All clinically acquired data were normally distributed. Continuous variables were presented as mean ± standard deviation. Categorical data were reported as frequencies and percentages. A two-sample independent *t*-test was used to compare continuous variables. The *χ*^2^ test was used for categorical data. A paired *t*-test was used to compare sex differences in invasive PCWP vs. CMR-modelled PCWP.

Within the derivation cohort, multivariable linear regression (ENTER method) was used for each sex to generate partial correlation coefficients (using all other variables as covariates) for individual CMR metrics compared with PCWP measurement by RHC. Stepwise multivariable regression was then used to develop a sex-specific CMR-derived PCWP model. The sex-specific CMR model was applied to the validation cohort, and receiver operating characteristic analysis was performed to assess the diagnostic power of CMR-derived PCWP to detect raised RHC PCWP (defined a priori as >15 mmHg). Kaplan–Meier analysis and Cox proportional hazard model were used for multivariable analysis of prognosis. Statistical analysis was performed in SPSS version 22 (IBM, Chicago, USA) and confirmed in MedCalc (MedCalc Software, Ostend, Belgium version 19.1.5). Unless otherwise stated, all statistical tests were two tailed, and a *P*-value of <0.05 was deemed significant. Graphical representations were created in OriginLab (OriginLab Corporation, Northampton, MA, USA).

## Results

### Study population (derivation cohort)

A total of 835 participants were included within the derivation cohort, and 60% were female. The patient characteristics are summarized in *Table 1*. Both sexes had similar ages (66 ± 13 years, *P* = 0.84). Females exhibited a significantly lower mean body surface area (BSA) than males (1.8 ± 0.2 vs. 2.0 ± 0.2 m^2^, *P* < 0.0001). Females had higher systolic blood pressure than males (146 ± 28 vs. 140 ± 24 mmHg, *P* < 0.001). Females displayed a slightly higher mean heart rate than males (72 ± 15 vs. 70 ± 16 b.p.m., *P* = 0.02). There was a lower prevalence of chronic obstructive pulmonary disease in females than in males (9% vs. 15%, *P* = 0.005). As expected, HFpEF was more common in females than males (62% vs. 40%, *P* < 0.001), while males had more HF with mid-range EF (7% vs. 2%, *P* < 0.001). There was no significant difference between invasive mean PCWP between females and males (14.0 ± 6 vs. 13.7 ± 6 mmHg, *P* = 0.52).

**Table 1:** Patient characteristics, cardiac magnetic resonance imaging assessment, and pulmonary capillary wedge pressure assessment stratified by sex

	Female sex (*n* = 497)	Male sex (*n* = 338)	*P*-value
Age (years)	66 ± 13	66 ± 13	0.84
Body surface area (m^2^)	1.8 ± 0.2	2.0 ± 0.2	<0.0001
Heart rate (b.p.m.)	72 ± 15	70 ± 16	0.02
Chronic obstructive pulmonary disease, *n* (%)	45 (9)	52 (15)	0.005
Heart failure preserved ejection fraction, *n* (%)	306 (62)	136 (40)	<0.0001
Heart failure mid-range ejection fraction, *n* (%)	10 (2)	22 (7)	<0.001
Heart failure reduced ejection fraction, *n* (%)	12 (2)	11 (3)	0.47
Other, *n* (%)	169 (34)	169 (50)	<0.0001
**Cardiac magnetic resonance imaging data**			
Heart rate (b.p.m.)	77 ± 14.0	74 ± 14	0.0004
Systolic blood pressure (mmHg)	146 ± 28	140 ± 24	<0.001
Diastolic blood pressure (mmHg)	78 ± 12	78 ± 14	0.32
Left ventricular end-diastolic volume (mL)	103 ± 30	121 ± 43	<0.0001
Left ventricular end-systolic volume (mL)	32 ± 17	45 ± 24	<0.0001
Left ventricular stroke volume (mL)	71 ± 22	76 ± 27	0.001
Left ventricular ejection fraction (%)	69 ± 11	63 ± 11	<0.0001
Left ventricular mass (g)	84 ± 24	118 ± 36	<0.0001
Left atrial volume (mL)	75 ± 39	82 ± 49	0.01
Right ventricular end-diastolic volume (mL)	131 ± 52	173 ± 67	<0.0001
Right ventricular end-systolic volume (mL)	71 ± 40	106 ± 53	<0.0001
Right ventricular stroke volume (mL)	60 ± 24	67 ± 29	<0.001
Right ventricular ejection fraction (%)	47 ± 13	40 ± 13	<0.0001
**PCWP assessment**			
Invasive PCWP (mmHg)	14.0 ± 6.0	13.7 ± 6.0	0.5
Generic CMR-derived PCWP	13.0 ± 3.0	14.7 ± 4.0	<0.001
Sex-specific CMR-derived PCWP	14.1 ± 3.0	13.8 ± 4.0	0.3

### Cardiovascular magnetic resonance evaluation (derivation cohort)

**Figure 2:**
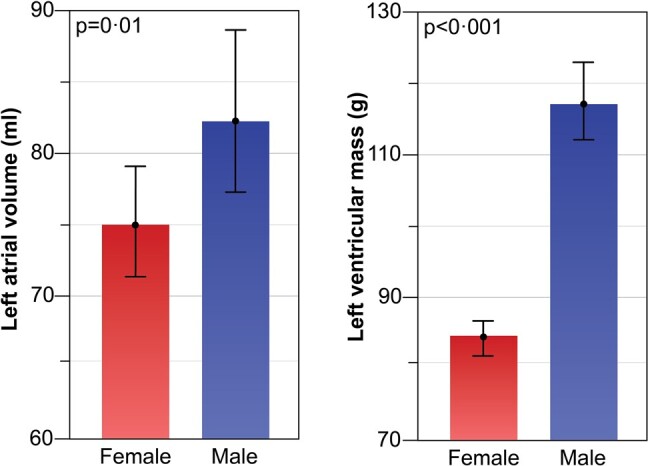
Within the derivation cohort (*n* = 835), left atrial volume and left ventricular mass were significantly lower in females than in males.

Differences between males and females in CMR volumetric and functional assessment are detailed in *Table 1* for the derivation cohort. Females had lower LVEDV and LVESV, resulting in smaller LVSV and higher LVEF, than males. Males demonstrated higher LVM and LAV (*Figure 2*). In the RV, females had a lower RVEDV, RVESV, RVSV but a higher overall RVEF. Generic CMR-derived PCWP^14^ values were significantly higher in males (14.7 ± 4.0 vs. 13.0 ± 3.0 mmHg, *P* < 0.001) compared to females.

### Cardiovascular magnetic resonance link to invasive assessment of pulmonary capillary wedge pressure (derivation cohort)

**Figure 3:**
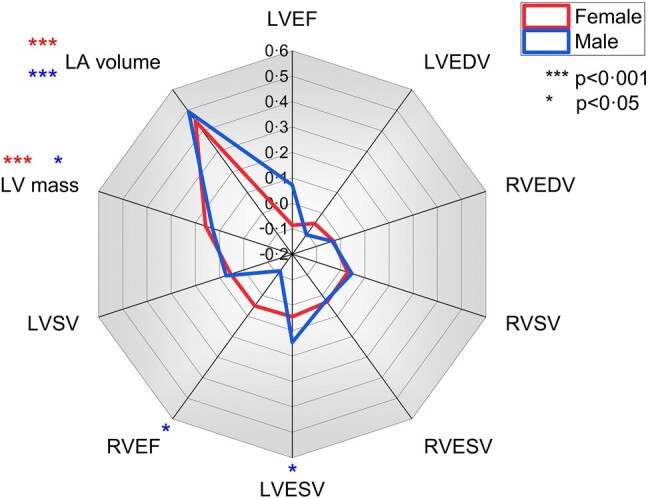
Radial line plot demonstrating that within the derivation cohort (*n* = 835), left atrial volume and left ventricular mass were significantly associated with invasively measured pulmonary capillary wedge pressure during partial correlation analysis.

**Figure 4:**
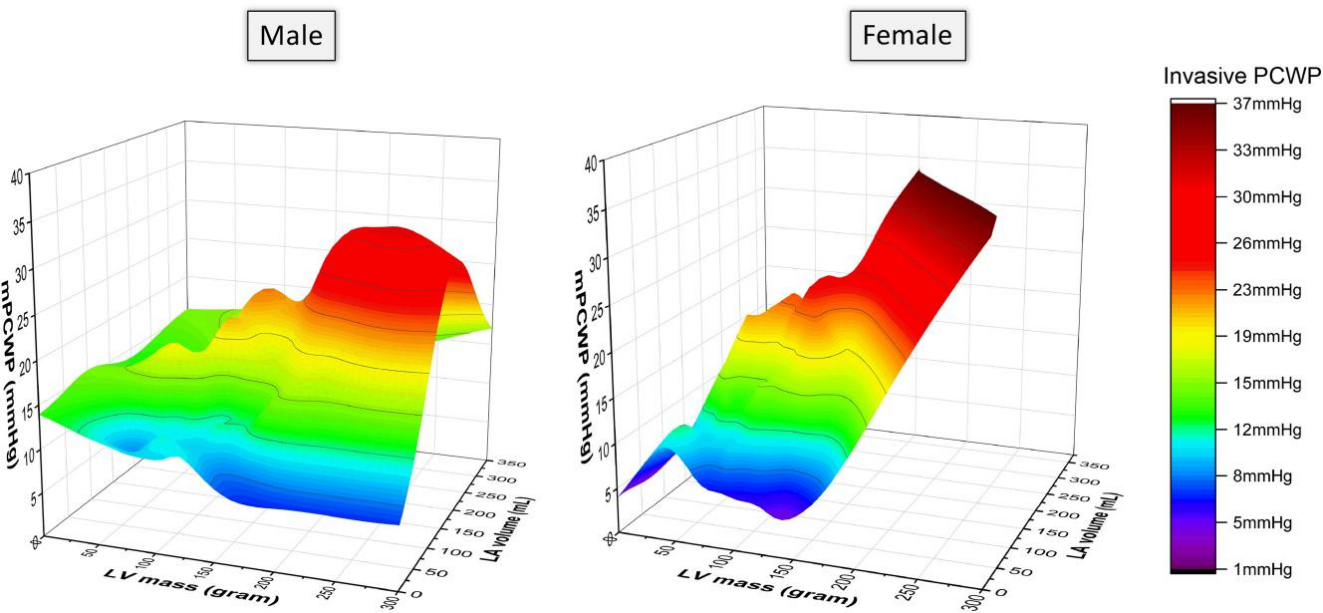
3D surface plot showing sex-specific profiles in the relationship between left ventricular mass, left atrial volume, and invasive mean pulmonary capillary wedge pressure within the derivation cohort (*n* = 835).

Out of all the CMR variables, CMR-derived LAV was most strongly associated with mean invasive PCWP in both sexes (*Table 2*). Due to the interdependence of CMR variables with each other, we further investigated the independent association of each parameter to invasive PCWP using ENTER multiple regression. Here again, LAV (females: partial *r* = 0.45, *P* < 0.001; males: partial *r* = 0.49, *P* < 0.001) and LVM (females: partial *r* = 0.16, *P* < 0.001; males: partial *r* = 0.13, *P* = 0.02) were independently associated with invasive PCWP in both sexes, even after factoring in all other variables as covariates (*Figure 3*). The pattern of association between LVM, LAV, and invasive PCWP is sex specific, as demonstrated in *Figure 4*.

**Table 2:** Correlation between cardiovascular magnetic resonance variables and mean invasive pulmonary capillary wedge pressure in males and females within the derivation cohort

	Females		Males	
	*R*	*P*-value	*R*	*P*-value
Left ventricular end-diastolic volume	0.31	<0.0001	0.36	<0.0001
Left ventricular end-systolic volume	0.22	<0.0001	0.30	<0.0001
Left ventricular stroke volume	0.24	<0.0001	0.30	<0.0001
Left ventricular ejection fraction	−0.06	0.2	−0.06	0.3
Left ventricular mass	0.29	<0.0001	0.33	<0.0001
Left atrial volume	0.54	<0.0001	0.58	<0.0001
Right ventricular end-diastolic volume	0.21	<0.0001	0.11	0.05
Right ventricular end-systolic volume	0.11	0.01	0.02	0.74
Right ventricular stroke volume	0.27	<0.0001	0.21	0.0001
Right ventricular ejection fraction	0.07	0.1	0.10	0.08

### Sex-specific cardiovascular magnetic resonance–derived pulmonary capillary wedge pressure (derivation cohort)

Sex, LAV, and LVM were used as the three input variables in stepwise multivariable regression, with age as a weighted variable. The following sex-specific equation was determined with goodness of fit (*R*-value) of 0.571:


CMRPCWP=5.7591+(0.07505*LAV)+(0.05289*LVM)–(1.9927*sex)[female=0;male=1].

Given the systematic difference in BSA between males and females, indexed volumetrics and BSA directly were also tried in the model (see Supplementary material online, *Appendix S1*). However, indexed LAV and LVM values gave a worse goodness of fit (*R* = 0.470) and did not remove the independent influence of sex; hence, the non-indexed equation was selected.

### Internal cross-validation (derivation cohort)

**Figure 5:**
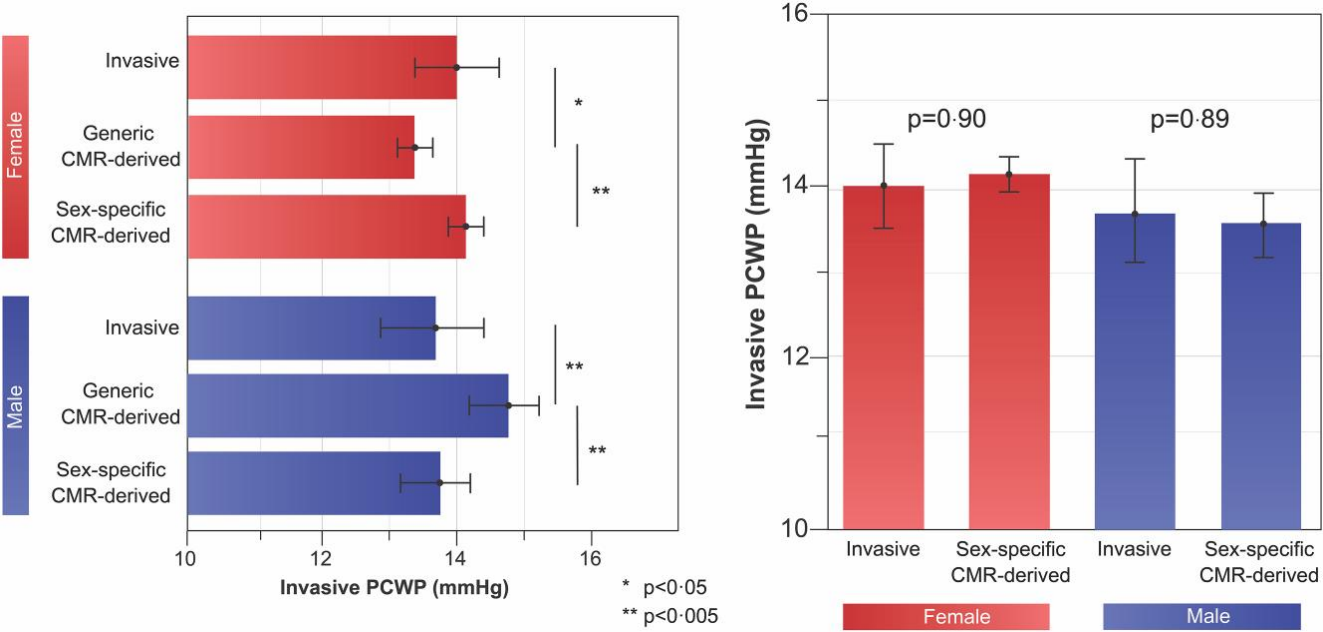
Within the derivation cohort, the difference in assessment between invasive and generic cardiovascular magnetic resonance–derived pulmonary capillary wedge pressure was significant across both sexes. There was no statistical difference between the invasive and sex-specific cardiovascular magnetic resonance–derived pulmonary capillary wedge pressure.

In multiple stepwise regression, the novel sex-specific model retained its independent association with invasively measured PCWP, whereas the previously used generic CMR-derived model was excluded (beta = 1, standard error = 0.005, *P* < 0.0001, partial *r* = 0.57). In paired comparisons between invasive and generic CMR-derived PCWP, the generic equation underestimated PCWP in females (14.0 ± 6.0 vs. 13.0 ± 3.0 mmHg, *P* = 0.01) and overestimated PCWP in males (13.7 ± 6.0 vs. 14.7 ± 4.0 mmHg, *P* < 0.001, *Figure 5*). In contrast, PCWP did not differ significantly between invasive assessment and sex-specific CMR equation.

### Validation within an external heart failure cohort

**Figure 6:**
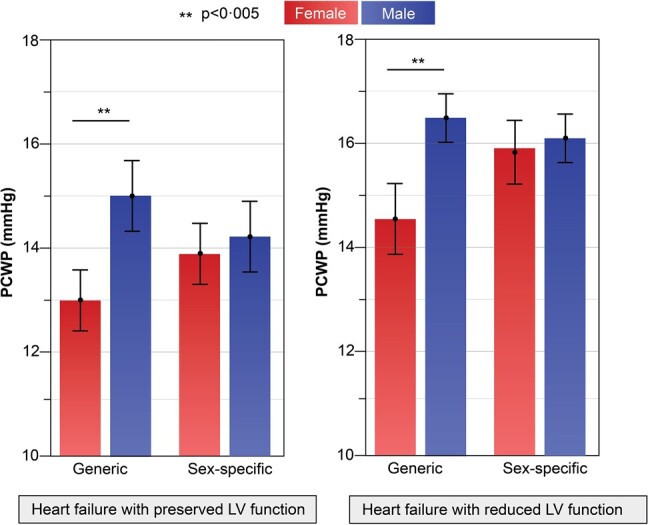
Within the validation cohort, pulmonary capillary wedge pressure assessment using the sex-specific model resulted in comparable mean pulmonary capillary wedge pressure between sexes, which was not the case using the generic cardiovascular magnetic resonance–derived pulmonary capillary wedge pressure model. This finding was sustained in both heart failure with preserved and reduced LV function.

The above equations were applied to the validation cohort (*n* = 454, 36% female) to estimate PCWP. From this population, 52% (*n* = 240) had HFpEF, and the rest had HFrEF (48%, *n* = 214). In both HFpEF and HFrEF, the generic CMR equation demonstrated a significant difference in derived PCWP between males and females (*P* < 0.001, *Figure 6*). However, when utilizing sex-specific CMR-derived PCWP values, no statistically significant difference was observed between females and males (14.1 ± 3.0 vs. 13.8 ± 4.0 mmHg respectively, *P* = 0.3), indicating resolution of the previous sex-specific bias in the generic equation (*Figure 6*).

### Survival analysis for heart failure hospitalization (validation cohort)

**Figure 7:**
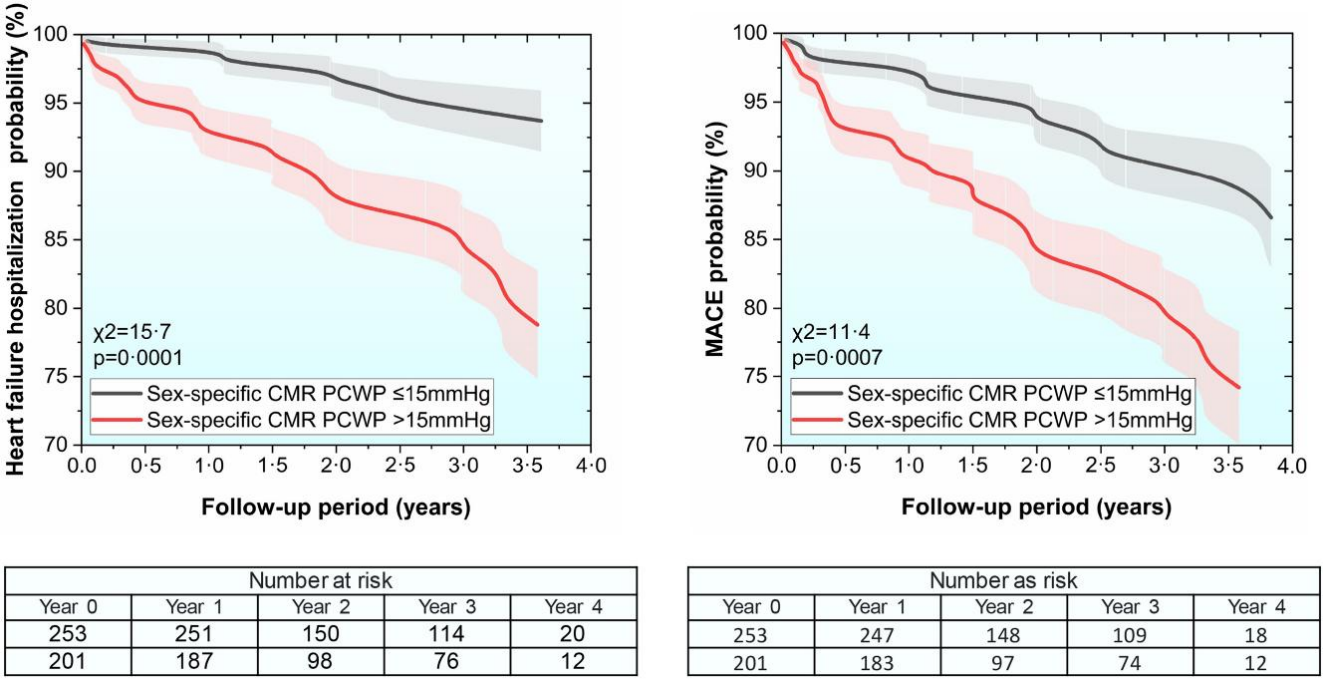
Kaplan–Meier survival curves demonstrate the prognostic significance of sex-specific cardiovascular magnetic resonance pulmonary capillary wedge pressure assessment at a threshold of 15 mmHg within the validation cohort (left panel—heart failure hospitalization; right panel—major adverse cardiovascular event).

During a mean follow-up period of 2.4 ± 1.2 years, 38 (8.4%) patients were hospitalized with decompensated HF. In multivariable Cox proportional hazard regression, factoring in both raised generic and sex-specific CMR-derived PCWP (>15 mmHg), only the sex-specific model demonstrated independent association to HF hospitalization [beta = 1.4, standard error = 0.37, *P* = 0.0002, hazard ratio (HR) 3.9, 95% confidence interval (CI) 1.9–8.0]. In Kaplan–Meier analysis, sex-specific CMR-modelled PCWP was predictive of survival for both males and females (*χ*^2^ = 15.7, *P* = 0.0001, *Figure 7*).

### Survival analysis for major adverse cardiovascular event (validation cohort)

During a mean follow-up period of 2.4 ± 1.2 years, 56 (12.3%) patients had MACE. In multivariable Cox proportional hazard regression factoring in both raised generic and sex-specific CMR-derived PCWP (>15 mmHg), only the sex-specific model demonstrated independent association to MACE (beta = 0.92, standard error = 0.23, *P* = 0.001, HR 2.5, 95% CI 1.4–4.3). Cut-off values for CMR PCWP were not different between HFpEF or HFrEF groups (see Supplementary material online, *Appendix S2*). In Kaplan–Meier analysis, sex-specific CMR-modelled PCWP was predictive for MACE (*χ*^2^ = 11.4, *P* = 0.0007, *Figure 7*).

Cardiovascular magnetic resonance PCWP remained predictive when LVEF was also factored into the analysis for both MACE and HF hospitalizations, suggesting that it was an independent outcome predictor across all HF classifications (see Supplementary material online, *Appendix S3*).

## Discussion

The present study investigates the impact of sex on cardiac imaging methods of HF assessment. Women are exposed to different biological, psychosocial, and socioeconomic risk factors compared with men, particularly on a global scale,^1^ and it is therefore important to ensure measurement validity in both sexes. A novel sex-specific equation for LVFP estimation is validated and demonstrates superior performance. The previously derived generic CMR-derived PCWP equation overestimated PCWP in males and underestimated PCWP in females: potentially resulting in over- and under-diagnosis, respectively, especially in early or borderline disease. The novel CMR sex-specific PCWP equation resolves this issue. Both during internal cross-validation and validation within a large external cohort of HF patients, the sex-specific CMR-derived PCWP model had superior prognostic capabilities for HF hospitalization and MACE.

The elegance of previous CMR-derived PCWP estimation has been that it uses two robust anatomical measurements: LVM, a marker of afterload, and LAV, a marker of preload, on the LV. However, it is also established that both these CMR-derived parameters are lower in females than in males,^27^ reflecting innate biological differences not necessarily related to loading conditions. It is therefore logical that these prior differences should be accounted for to allow dynamic LVM and LAV variations to accurately reflect the underlying haemodynamic state. There are well-accepted differences in body habitus between males and females at a population level, and indexing is often used to improve comparability of single volumetric measurements.^28^ There are however concerns about the best measure to use for indexing, particularly in certain populations such as obesity.^29–31^ Obesity is common in HFpEF and cardiovascular conditions in general.^32^ In the present study, we did not find improvement in the model for BSA indexed values, and sex continued to be an independent modifier despite adjustment. This might suggest that biological sex is more important than simple differences in body size. In composite measures with multiple indexed values, the additional variable might also introduce measurement error greater than any added value it provides for prediction.

The ASPIRE registry represents a mixed population with HF, pulmonary disease, and pre-capillary pulmonary hypertension and includes a range of normal and abnormal PCWPs. This heterogeneity is ideal for the derivation of a CMR PCWP allowing for a range of filling pressures in both cardiac and non-cardiac disease, with similar clinical symptoms, with high external validity. The Leeds registry provides a well phenotyped cohort of ischaemic and non-ischaemic cardiomyopathy, reflective of everyday practice, and enhancing outcome generalizability.

Left atrial volume provides a sensitive marker of underlying haemodynamics. In normal physiological conditions, an increase in preload (such as during exercise or volume overload) leads to an increase in LAV. This distension results in more forceful LA contraction, maintaining efficient blood flow into the LV.^33^ However, in pathological conditions such as HF, the relationship between preload and LAV can be altered. For instance, in HFpEF, the LV becomes stiff and less compliant, leading to increased LVFP.^34^ This increased pressure is reflected into the left atrium, causing LA enlargement.^35^ Furthermore, studies have shown that acute changes in preload can also affect LAV.^20^ Left atrial function, at both rest and during exercise stress, has emerged as the strongest predictor of cardiovascular outcomes in the HFpEF stress trial.^36^ In a sub-analysis of this study, the resting LAV was different between males and females, and the changes in LA EF and LA filling reserve were significantly impaired in women with HFpEF.^37^ The LV filling pressure has also been shown to increase significantly in HFpEF patients with an EF >60% with exercise, with this subgroup likely reflecting a hypercontractile state secondary to high afterload.^38^ The present study adds weight to the concept of left atrial disease and elevated intracardiac pressures being central to HFpEF physiology in this large outcomes cohort.

This is also the first study that explores the associations of invasively measured PCWP with both left and right heart CMR-derived parameters. Interestingly, we find that LAV and LVM remain the most predictive variables in females. In males, RVEF and LVESV also showed some minor association. Left ventricular hypertrophy (LVH) is a complex adaptive response to increased afterload,^39^ predominantly occurring in conditions such as systemic hypertension and aortic stenosis,^40,41^ entailing a cascade of cellular and molecular alterations to accommodate the augmented mechanical workload.^42^ Myocyte hypertrophy, a hallmark of LVH, ensues from cellular signalling pathways leading to enhanced contractile protein synthesis, thereby augmenting the force-generating capacity of cardiomyocytes.^43^ Activation of growth factors such as angiotensin II and transforming growth factor-beta (TGF-β) contributes to fibroblast proliferation and extracellular matrix remodelling.^44^ Fibrosis promotes myocardial stiffness and structural alterations—both of which are the hallmarks of LV diastolic dysfunction. In many instances, load-induced LVH is treatable, and hence, the CMR-derived PCWP is a novel therapeutic biomarker in patients with HF.^45^

The present work is unable to comment specifically on the clinical management for HFpEF, as its scope was diagnostic. However, it is hypothesis generating that treatments, such as SGLT2 inhibitors, might be more efficacious when personalized to those with raised filling pressures. Other works sub-phenotyping HFpEF have suggested similar strategies for this heterogeneous group.^38^

### Limitations

In the derivation cohort, there is the possibility of selection bias as this study was undertaken at a tertiary centre that took referrals for RHC assessment, potentially limiting recruitment of the very elderly or frail. However, it is noteworthy that our study stands as one of the largest investigations that encompassed a diverse cohort of patients, aiming to explore PCWP using CMR. All patients included were clinically stable seeking care for breathlessness in an outpatient setting and did not encompass acute HF patients; hence, it remains to be seen if the methods established here are applicable in the latter situation.

The study was conducted in a broad cohort of HF patients with predominantly ischaemic or non-ischaemic cardiomyopathy. We have not sought to validate the equation in specific aetiologies of HF where study power would be much reduced. The results should not therefore be applied to significant valvular heart disease, as such patients were excluded from both the derivation and validation cohorts, and because there might be different courses of LAV and LVM changes in these patients, particularly in regurgitant lesions. The sex-specific CMR PCWP is more dependent on LAV than LVM; therefore, we hypothesize it would be useful in infiltrative and hypertrophic cardiomyopathies, where there is increasing evidence that changes in LAV are both part of the progression of disease and also prognostic.^46–51^ Further studies are required to validate the sex-specific CMR PCWP specifically in each of these aetiologies. The results of this study should also not be applied to athletes who have a higher LVM and LAV due to positive remodelling, compared to adverse remodelling in patients with high PCWP, without further validation.

The RHC and CMR data were obtained at rest, rather than with exercise stress, in this study. There is increasing evidence of abnormal physiological response of cardiac function and volumetrics to exercise in HFpEF,^52^ particularly in women.^37^ It is therefore possible that such cases would be missed or inconclusive using the present method, and that exercise-related measures might improve prediction of PCWP. However, adding exercise stress into routine CMR acquisition is more technically challenging, time consuming, and expensive, with most CMR imaging worldwide conducted in the resting state only. The present approach would potentially identify cohorts with raised PCWP at rest, allowing resources for exercise stress to be focused on marginal cases.

Sex assigned at birth was utilized in this study rather than karyotype or gender identity; hence, the results may not be applicable in intersex individuals or those who have chosen an alternative gender. There has been a marked increase in the number of young people identifying a transgender identity.^53^

## Conclusions

A sex-specific CMR-modelled LVFP improves the precision of PCWP estimation and prognostic performance in patients with HF.

## Supplementary Material

oeae038_Supplementary_Data

## Data Availability

Deidentified data underlying this article will be shared on reasonable request to the corresponding author for further academic study following publication. As this is a multicentre study, agreement would be required from all contributing centres.

## Lead author biography

Dr Pankaj Garg is an associate professor in cardiovascular medicine at the University of East Anglia, Norwich, UK, and honorary consultant cardiologist at Norfolk and Norwich University Hospitals. He graduated in medicine with honours from I.M. Sechenov (2003) and became a member of the Royal College of Physicians in 2007. After completing his BHF-funded PhD at Leeds (2017), he was awarded the NIHR Clinical Lectureship programme at the University of Sheffield. In 2020, he was awarded Wellcome Trust Clinical Research Career Development Fellowship. His research focus is cardiovascular haemodynamics and cardiac magnetic resonance imaging. His ambition is to discover and advance a better understanding of the interface between cardiovascular flow and cardiac remodelling. He has received 20 national/international awards and has published >185 peer-reviewed papers.
