# Supplementary material for: Sex-specific cardiac magnetic resonance pulmonary capillary wedge pressure
Source: Eur Heart J Open. 2024 May 15;4(3):oeae038. doi: 10.1093/ehjopen/oeae038 (PMC11095051; doi:10.1093/ehjopen/oeae038)
Supplement: oeae038_Supplementary_Data [file oeae038_supplementary_data.docx]

**SUPPLEMENTARY MATERIAL**

**Appendix 1**

We studied the impact of indexing the LA volume and LV mass to the body surface area (BSA) on the sex-specific CMR PCWP equation. Using indexed values, the equation was:

**CMR PCWP = 0.053 + (0.412*LAVi) + (0.139*LVMi) – (0.135*sex) [female = 0; male = 1]**

With LAVi (p<0.001), LVMi (p<0.001) and sex (p=0.046) remaining significant as independently predictive values. The R-value for model goodness of fit was 0.470, which was less than use of non-indexed values. An alternative strategy where BSA was included in the model with non-indexed values resulted in the same CMR PCWP equation as presented in this study, as BSA was removed from the model (P=0.06). Hence indexing to BSA neither improves the model nor removes the effect of accounting for biological sex. This suggests biological sex is a greater modifier than accounting for body size alone.

**Appendix 2**

We checked that the CMR PCWP cut off points were similar when patients were dichotomised into either HFpEF or HFrEF groups using ROC analysis. In HFpEF the AUC for CMR PCWP was 0.811 (95% CI 0.772-0.847, P<0.0001) and the Youden index cut off was >14.7mmHg. For the HFrEF group, the AUC was 0.873 (95% CI 0.668-0.974, P<0.0001) and the Youden index cut off was >14.1mmHg. Both groups therefore have similar cut off points within 1mmHg, and both are extremely similar to the accepted 15mmHg cut off for RHC.

**Appendix 3**

To investigate whether CMR PCWP outperformed LVEF across the cohort we allowed both the LVEF and the CMR PCWP >15mmHg criteria to be co-variates in a Cox proportional hazards regression model weighted for age in the validation cohort. Survival curves were obtained as follows:


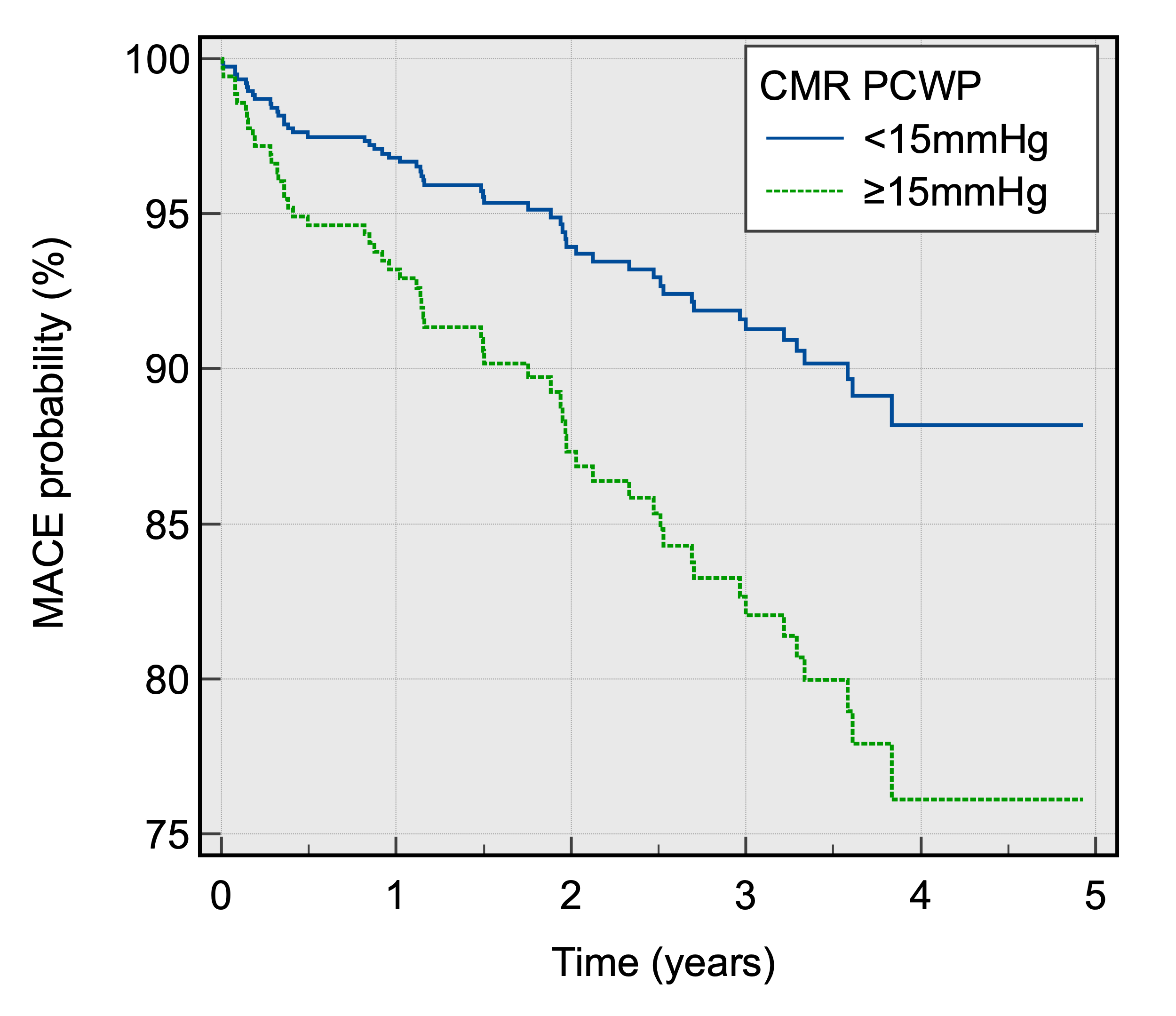


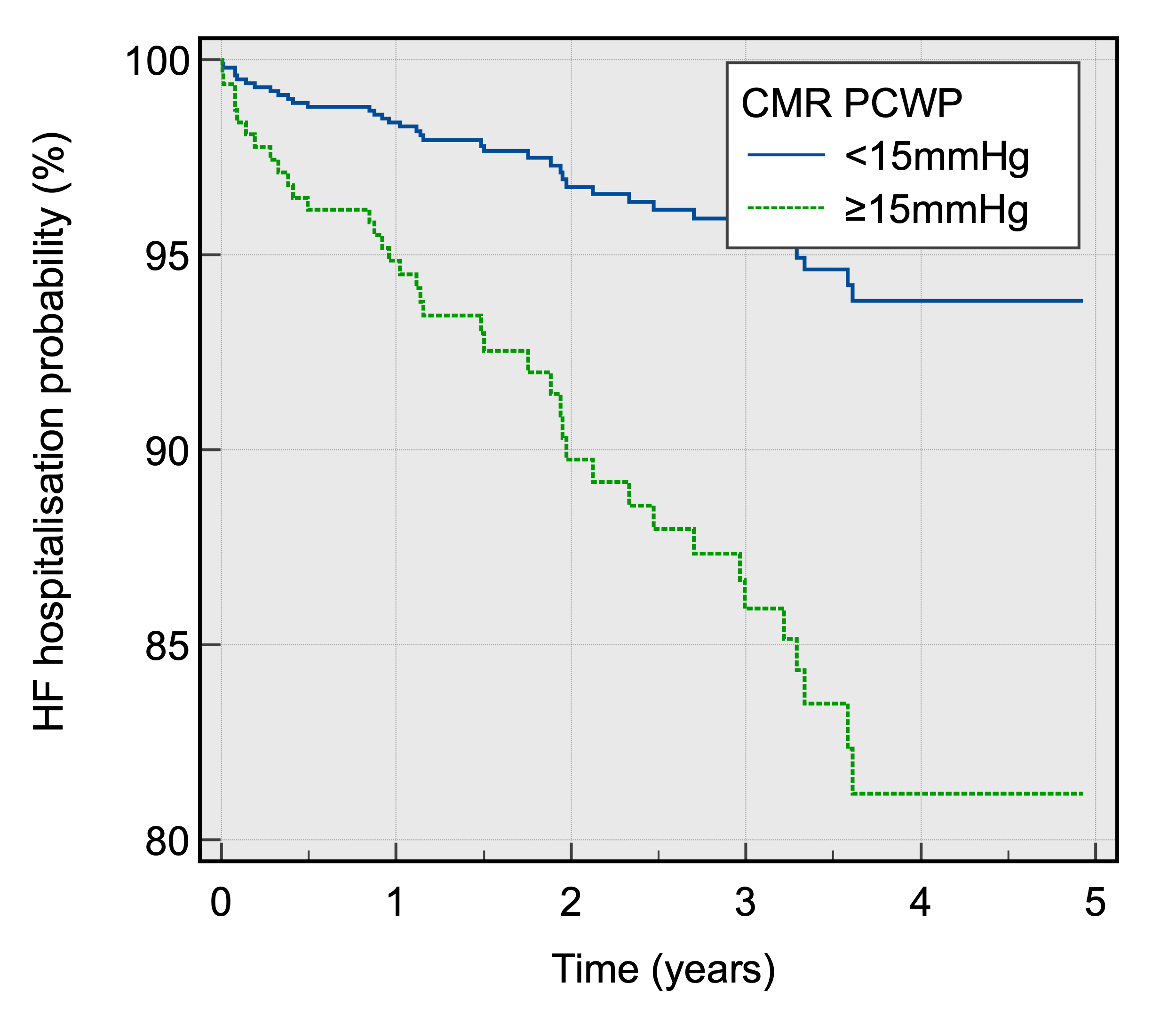


Both LVEF and CMR PCWP >15mmHg remained significant in the model. For MACE, the HR for LVEF was 0.97 (95% CI 0.9480-0.9926, p=0.0096) and for CMR PCWP was 2.17 (95% CI 1.1990 to 3.9149, p=0.010). For HF hospitalisations the HR for LVEF was 0.97 (95% CI 0.9453-0.9994, p=0.046) and for CMR PCWP was 3.27 (95% CI 1.5253-6.9995, p=0.0023). Therefore, both LVEF and CMR PCWP appear to be independently useful in outcome prediction for both MACE and HF hospitalisation. However, the effect size is numerically greater for CMR PCWP.
